# Sepsis in the Emergency Department: Different Faces, Same Fate? A Sex-Based Prognostic Analysis

**DOI:** 10.3390/ijms27093753

**Published:** 2026-04-23

**Authors:** Francesco Pepe, Maria Antonella Pinelli, Simona Giuliante, Francesca Sabatino, Lorenzo Sari, Silvia Visintainer, Lara Fuciarelli, Francesca Innocenti

**Affiliations:** High Dependency Unit, Careggi University Hospital, 50134 Florence, Italyinnocenti.fra66@gmail.com (F.I.)

**Keywords:** emergency department, sex-specific characteristics, sepsis

## Abstract

We aimed to evaluate the clinical characteristics of male and female patients with sepsis and to investigate their potential sex-specific differences in the prognostic weight. Retrospective analysis, including all patients with sepsis admitted to the Emergency Department High-Dependency Unit, between June 2008 and October 2025. Clinical data were collected using a standardized template. The endpoints were day-7 and day-28 mortality rates. The study population included 1527 patients, with a mean age of 74 ± 13 years, and female sex comprising 44%. Women were older (77 ± 13 vs. 73 ± 13 yrs, *p* ≤ 0.001), with a lower prevalence of cancer (19% vs. 27%, *p* = 0.009) than men. Conversely, a pulmonary (34% vs. 66%) or abdominal (40% vs. 60%) sepsis source was more frequent in men (both <0.05). The SOFA score was significantly lower in women than in men (5 [3–6] vs. 5 [3–7], *p* < 0.05), whereas day-7 and day-28 mortality rates were 11% and 22%, respectively, in both sexes. Multivariate analysis identified several independent predictors of 7-day mortality for both sexes. Women and men showed similar clinical characteristics and short- and medium-term mortality, despite a lower grade of organ dysfunction among women.

## 1. Introduction

Sepsis is one of the most common diagnoses in the Emergency Department (ED), and has recently been declared a time-dependent disease by the World Health Organization, as the early identification and treatment may significantly improve the outcome [1]. One of the key issues in the research field on the assessment and treatment of patients with sepsis is the persistent inclusion of undifferentiated patients in most recent trials. This may prevent potentially useful therapies from showing a prognostic advantage, possibly because they were not employed in the correct patient population [2,3]. A better characterization of patients with sepsis is needed to identify subgroups that can benefit from targeted treatment, both for hemodynamic resuscitation and management of the inflammatory storm.

From this perspective, sex is a significant feature. Sepsis is a disease characterized by the disruption of several components of the immune response, which shows various sex-specific characteristics. These differences may be explained by genetic, bio-humoral, and environmental factors, demonstrated in clinical and experimental settings. In women, increased expression of anti-inflammatory mediators could help improve control of the inflammatory response. Similarly, estrogens have been shown to have immunomodulatory effects and to enhance immune cell activity, while testosterone has the opposite effect. Differences related to sex-specific patterns of comorbidities, health behaviors, and a heterogeneous distribution of infection sources complete the picture. However, the extent to which these differences influence the clinical presentation and outcomes of female and male patients with sepsis remains unclear [4]. Epidemiologically, men have a higher overall prevalence of sepsis compared to women. This disparity is further characterized by a higher incidence of pulmonary sepsis in men, whereas urinary sepsis is more common among women. A recent meta-analysis (Antequera) included 13 studies, revealing highly heterogeneous results. The study populations ranged from 305 to over 27,000 patients, mostly assessed during ICU stays, with a small proportion evaluated in the ED. Five studies reported higher mortality in women than in men, one showed higher mortality in men, and the remaining studies did not find any significant difference. Two very large studies published in 2025 reported significantly lower mortality in women, at 12% vs. 13% and 26.4% vs. 27.6%, respectively. While these differences are statistically significant in large populations, they could disappear in smaller study groups and have limited clinical meaning. The reasons behind this discrepancy are speculative, and the actual causes of these results remain unknown [5,6].

The present study aimed to evaluate the clinical characteristics of female and male patients during the first 24 h after presentation to the ED for sepsis, and to investigate sex-specific differences in their prognostic weight.

## 2. Results

During the study period, 1576 patients were admitted to the HDU with a diagnosis of sepsis, but 49 of them were excluded due to incomplete clinical data. The final study population included 1527 patients, with a mean age of 74 ± 13 years and a female sex prevalence of 44%. Respectively, 695 and 832 patients were admitted in the first period, based on the 2001 SCCM/ESICM/ACCP/ATS/SIS criteria, and in the second period, based on Sepsis-3. The mean age (74 ± 14 vs. 75 ± 13 years), lactate level upon admission (3.3 ± 3.1 vs. 3.3 ± 3.4 meq/L) and after 24 h (2.0 ± 2.0 vs. 2.0 ± 2.2 meq/L), SOFA score upon admission (5 [3–6] in both subgroups) and after 24 h (5 [3–6] vs. 5 [3–6], all *p* = NS) were all similar in the 2 subgroups. The proportion of females was lower in the second period than in the first one (40% vs. 46%, *p* = 0.016).

### 2.1. Comparison Between Female and Male Patients

In Table 1, we reported previous medical conditions and sepsis source in the whole group and by sex. Female patients were older and showed a lower prevalence of a diagnosis of cancer than men; the sepsis source was significantly less frequently pulmonary or abdominal among women than men.

In Table S1, we compared vital signs and arterial blood gas parameters upon ED admission and after 24 h in male and female patients; respectively, 36 men and 31 women died in the first 24 h and were not included in the analysis at T24.

The hemodynamic profile, considering both individual parameters and systolic and diastolic shock indices, showed no significant differences. Women had significantly lower pH and higher CO_2_ levels, with no significant differences in lactate levels, bicarbonates, or respiratory rate. Hyperpnea is a common finding in septic patients, primarily due to cytokine storm. This pattern could suggest that, among women, this response is less frequent or less pronounced than in men. Consistent with the lower prevalence of a pulmonary source for the septic process, the Horowitz ratio was higher in women than in men at all evaluations.

**Figure 1:**
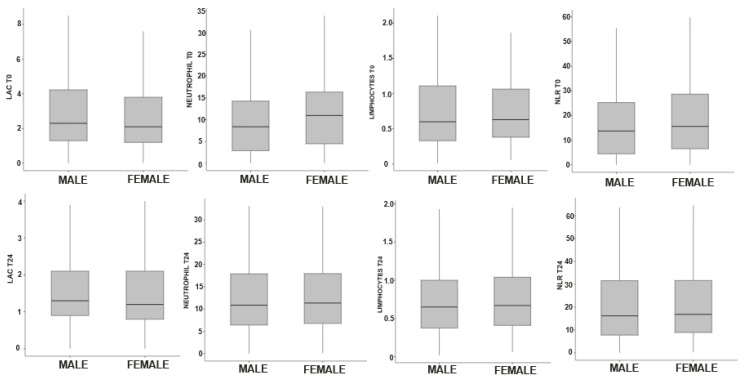
Inflammatory indices and lactate levels between male and female participants.

Among laboratory values, the only persistent difference over the 24 h of observation was a higher platelet count in women than in men. Inflammatory indices, like neutrophil, lymphocyte levels, or neutrophil/lymphocyte ratio, did not show any significant difference (Figure 1).

**Figure 2:**
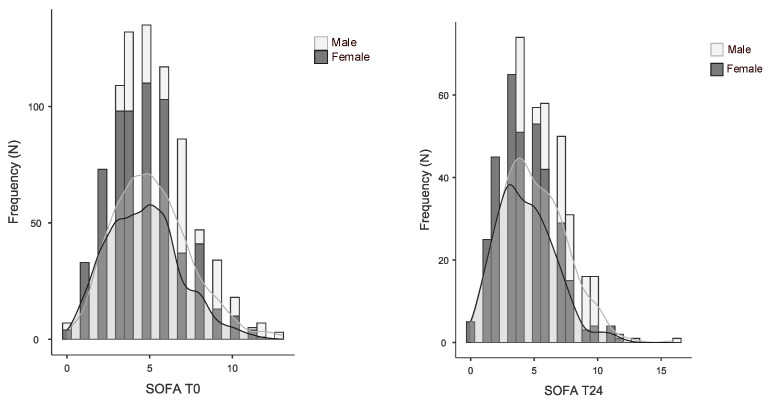
Distribution of the SOFA score between men and women. Both groups show a slight right-skewed distribution. The male cohort (light grey) exhibits a higher peak and a broader spread than the female group (dark grey).

The SOFA score was significantly lower in women than in men; Figure 2 shows the distribution of values in the two subgroups at T0 and T24.

**Figure 3:**
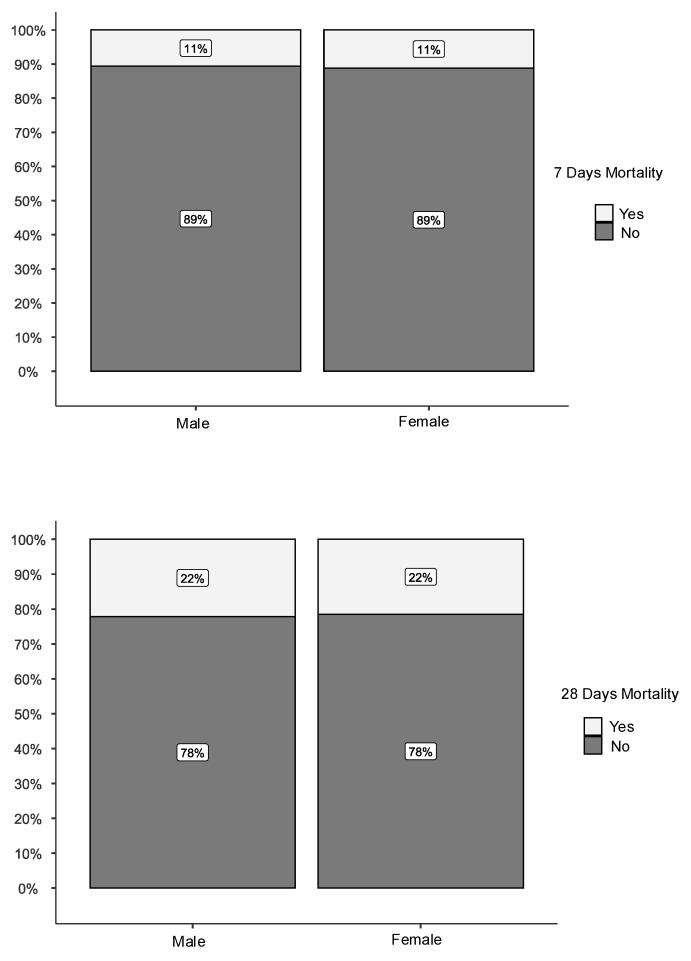
Day-7 and day-28 mortality.

As shown in Figure 3, despite a significantly lower SOFA score, day-7 and day-28 mortality rates were 11% and 22%, respectively, in both sexes.

In the univariate analysis, at T0 and T24, advanced age, high SOFA score, and lactate levels were all associated with increased mortality rates at day 7 and day 28, with independent prognostic significance confirmed by multivariate analysis. Conversely, sex did not demonstrate any prognostic value (Supplementary Material Table S2).

We performed a sensitivity analysis restricted to the 832 patients admitted under the Sepsis-3 criteria era (Supplementary Materials Table S5); the results were consistent with those obtained from the overall study population

### 2.2. Prognostic Determinants Among Women and Men

We analyzed prognostic determinants among women and men, separately. We selected several parameters, recognized as prognosticators among septic patients, and the results are shown in Table S3. At T0, among men and women, non-survivors had higher lactate levels and SOFA scores. At T24, compared to survivors, both male and female non-survivors showed more compromised hemodynamic status and a higher neutrophile/lymphocyte ratio (Supplementary Material Table S3).

At univariate analysis, advanced age, increased lactate levels, and high SOFA score were all associated with increased day-7 and day-28 mortality rates in both sexes. When inserted in a multivariate analysis for day-7 mortality rate (Table 2), in men all T0 and T24 variables maintained an independent predictive value. Conversely, in women, T0 lactate did not show an independent value in the multivariate model (*p* = 0.170).

Considering the day-28 mortality rate (Supplementary Materials Table S4), SOFA score, and age confirmed as strong independent predictors in both sexes. T24 Lac remained a significant independent predictor only in women (OR 1.26, 1.08–1.56, *p* < 0.001) while its effect was not significant in men (*p* = 0.160), after adjusting for other covariates.

To formally assess whether the sex-specific prognostic value of lactate was attributable to differences in subgroup sizes between males and females, an interaction test (sex × Lactate) was performed within the multivariable model. The interaction term was not statistically significant in all iterations with T0 and T24 values for both endpoints.

We performed a Cox proportional hazard survival analysis, stratified by sex and age.

**Figure 4:**
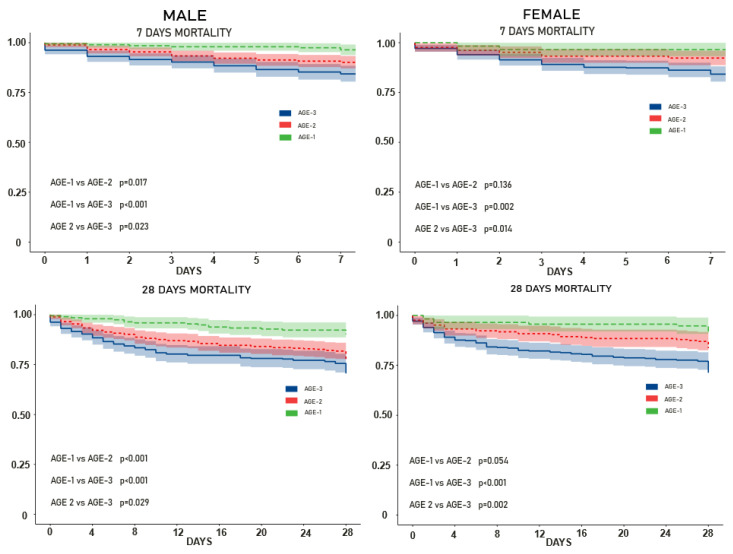
Cox survival curves for 7-day and 28-day mortality by sex and age subgroups. Dashed lines represent survival probabilities, and shaded areas indicate the 95% confidence intervals. Survival decreases significantly with increasing age group (AGE-1 > AGE-2 > AGE-3), with *p*-values for pairwise comparisons reported in each panel.

For women, only age ≥80 was associated with significantly higher 7-day mortality, whereas in men the survival curves suggested a progressive worsening with age, although not all pairwise subgroup comparisons reached statistical significance (Figure 4). The same pattern was observed in 28-day mortality.

## 3. Discussion

In a large population of patients admitted to the ED for sepsis or septic shock and managed for the first 24 h in an ED-HDU, we observed similar clinical characteristics regardless of sex. Short- and medium-term mortality rates were similar, despite a lower grade of organ dysfunction among women, as reflected by a lower SOFA score. Prognosticators were similar in men and women, both at ED presentation and after the first 24 h.

In the era of precision medicine, growing attention is being paid to the role of sex in the outcomes of patients with sepsis, yet results on this topic remain at least partially conflicting. Two recent meta-analyses, conducted in 2017 and 2023, did not reach clear conclusions. Papathanassoglou and coll. described a small survival disadvantage among women but underlined the paucity of well-designed studies and considerable heterogeneity among those available, making it impossible to draw strong conclusions on this topic [7]. Six years later, Antequera and coll. reported very low-certainty evidence for a possible prognostic advantage of female sex [5]. These conflicting results are coupled with the limited representation of female patients in both experimental models of sepsis and clinical trials, which hampers the ability to explain the potential mechanisms underlying these discrepancies.

In our study population, we examined various aspects of sepsis syndrome but found no significant sex-specific differences. The prevalence of prior medical conditions was similar, whereas women were less likely to present with sepsis of pulmonary origin, a source reportedly associated with a worse prognosis than other sources. The hemodynamic profile was comparable, with similarities persisting over the first 24 h, suggesting a similar response to early resuscitation. In a multivariate analysis that included known sepsis prognostic factors, sex was not independently associated with mortality.

In this framework, the only inconsistent finding was the mortality rate, despite women having a significantly lower SOFA score, suggesting that factors beyond organ damage might influence mortality in women. This phenomenon has been observed previously by Wernly and colleagues, who were unable to explain its underlying cause [8]. Conversely, in a small ICU population of septic patients, Jacobson and team found that the SOFA score at ICU admission was significantly higher in survivors than in non-survivors, but only among women, mainly due to differences in the coagulation SOFA sub-score [9]. Similarly, in a large ED population, Wanrooij and colleagues reported lower SOFA scores in women, who also exhibited lower mortality rates; however, female sex alone was not independently associated with reduced mortality [10]. Our study involved a population similar to previous research but included several new elements. We extended the evaluation to the first 24 h and incorporated the very short-term mortality rate, which had not been examined before. When assessing the prognostic value of parameters collected early in the ED, a short-term endpoint is especially important. Over time, complications may develop beyond the initial assessment that can influence outcomes. In addition to comorbidities and infection source, we analyzed inflammation parameters and performed separate multivariate regressions for males and females to determine whether known prognostic factors are equally effective across sexes.

We looked for other factors that might explain the excess mortality despite less organ damage, particularly inflammatory markers, but in this study group, these abnormalities were similar in women and men.

The host response to sepsis differs by sex because most immune cells are affected by sex hormones. Estrogens seem to sustain innate immune function mainly by decreasing monocyte–macrophage activation and reducing cytokine storms. Other possible mechanisms include increased neutrophil activity and dendritic cell maturation. Adaptive immunity might be supported through specific effects on CD4+ T-cell subsets [11]. During infections, bacteria are cleared more rapidly in females than in males, and this difference vanishes in ovariectomized animals. Additionally, females reportedly have lower immune reactivity than males, possibly as an adaptive response to pregnancy [12]. An opposing action of sex hormones on innate and adaptive immune responses has been reported, as estrogens support them, whereas androgens tend to have a suppressive effect. As a result, females generally exhibit a less intense inflammatory response than males. The second X chromosome may also contribute, as it contains multiple genes involved in both innate and adaptive immunity. Although much of its activity is inactivated, up to 23% of these genes remain active and help produce a stronger immune response. Finally, there is significant interaction between sex and steroid receptors, as they also interact with sex hormones in both men and women [13].

Despite this evidence, we did not observe significant sex-based differences in inflammatory parameters, and we question whether this discrepancy could be influenced by a complex interplay between sex and age. The immune response evolves with age, with upregulation of pro-inflammatory cytokines in both healthy and diseased elderly individuals. Healthy elderly people exhibit a subclinical, low-grade systemic pro-inflammatory state, known as “inflammaging” [14], triggered by the innate immune system and involving non-immune cells such as endothelial cells, adipocytes, and other senescent cells. This process raises plasma levels of several cytokines, including interleukin-6, tumor necrosis factor-alpha, interleukin-1beta, and other pro-inflammatory mediators [15]. The baseline imbalance between pro-inflammatory and anti-inflammatory responses creates an unfavorable starting environment. During sepsis, a harmful “cytokine storm” can develop, leading to greater organ dysfunction than in younger individuals. Because women in this study population were older than men, the sex-dependent protective effect may have been attenuated by the negative hyperinflammatory state associated with advanced age.

The present study has several limitations. The single-center, retrospective design inherently limits generalizability. We did not distinguish between sex and gender because, in this study population, the two terms were the same for all patients. We are aware that their distinction will gain further relevance in the coming year, but we can affirm that this was not the case for the patients included in this study.

## 4. Materials and Methods

This retrospective study included all patients admitted to the Emergency Department High-Dependency Unit at the University Hospital Careggi, Florence, Italy, from June 2008 to October 2025 with sepsis or septic shock.

The Local Ethics Committee (Toscana Area Vasta Centro) and the Institutional Review Board approved this study (n. OSS_28102).

Sepsis diagnosis was based on the 2001 SCCM/ESICM/ACCP/ATS/SIS criteria up to 2016 [16] and Sepsis-3 Criteria thereafter [17]. We exclude patients diagnosed by the first criterion, if they had an initial SOFA score < 2. Based on these criteria, we excluded 56 patients. The only exclusion criteria were age < 18 years and a positive COVID-19 test result.

Data was extracted from electronic medical records using a standardized template. We gathered information on previous medical conditions, vital signs, arterial blood gas parameters, and laboratory tests upon admission to the Emergency Department (ED, T0) and after 24 h (T24). Accordingly, the SOFA score was calculated at the same time points (T0 and T24) as in the original paper by Vincent et al. [18]. From 2018 onward, the leukocyte formula became available among urgent labs, allowing us to collect these data from medical records. The primary endpoints were mortality rates on day 7 and day 28.

We dichotomized age into tertiles in this population, with AGE-1 including those aged <65 years, AGE-2 those aged 65–79 years, and AGE-3 those aged ≥80 years.

### Statistical Analysis

Dichotomous variables were reported as absolute numbers and percentages, while continuous variables were reported as mean ± standard deviation or median and interquartile range, depending on their distribution. Dichotomous variables were compared using the Chi-square test with Bonferroni correction.

Candidate predictors for mortality were selected using LASSO (Least Absolute Shrinkage and Selection Operator) regularization to prevent overfitting and ensure model stability. All variables included in the final multivariate analysis (age, SOFA score, and lactate levels) were tested in sex-based subgroups and for both primary outcomes and demonstrated non-zero coefficients across all LASSO iterations.

Subsequently, univariate and multivariate analyses were conducted using Generalized Linear Models (GLM). To ensure the robustness of our inferences, we performed a non-parametric bootstrap (1000 samples, BCa methods) on all multivariate estimates. A *p*-value < 0.05 was considered statistically significant.

The analyses were performed using Jamovi 2.7.24.

## 5. Conclusions

In the era of precision medicine, considering sex when assessing prognosis and response to treatment in septic patients seems obvious, given the sex-specific characteristics of the immune response. The quality of the existing evidence and the presence of multiple discrepancies make it difficult to draw meaningful conclusions about the relationship between sex and sepsis-associated mortality. The outcomes appear similar, with the suspicion that the severity of organ damage plays a less significant role in women than in men. A complex interaction between sex and age could be one reason for the discrepancies observed across studies. As far as we know, sepsis-related mortality rates in either men or women might be influenced by unknown factors that need to be identified in future research.

## Figures and Tables

**Table 1:** Clinical characteristics of the study population.

	All (*n* = 1527)	Male (*n* = 858)	Female (*n* = 669)	p
Age (years)	74 ± 13	73 ± 13	77 ± 13	<0.001
COPD (%)	338 (22%)	199 (23%)	139 (21%)	0.339
CHD (%)	243 (16%)	135 (15%)	108 (16%)	0.140
Hypertension (%)	880 (58%)	496 (58%)	384 (58%)	0.869
Diabetes (%)	326 (28%)	190 (30%)	136 (26%)	0.183
CKD (%)	358 (23%)	219 (26%)	139 (21%)	0.063
Cancer (%)	358 (23%)	229 (27%)	130 (19%)	0.009
Septic shock (%)	691 (53%)	380 (52%)	311 (55%)	0.491
**Sepsis Source**				<0.001
Pulmonary (%)	307 (21%)	202 (66%)	105 (34%)	
Urinary (%)	431 (29%)	231 (54%)	200 (46%)	
Abdominal (%)	467 (31%)	278 (60%)	189 (40%)	
Skin and soft tissues (%)	122 (8%)	47 (39%)	65 (62%)	
Device (%)	31 (2%)	14 (45%)	17 (55%)	
Cardiac (%)	20 (1%)	12 (60%)	8 (40%)	
Unknown (%)	109 (7%)	51 (47%)	58 (53%)	

COPD: chronic obstructive pulmonary disease; CHD: coronary heart disease; CKD: chronic kidney disease.

**Table 2:** Univariate and multivariate analyses in men and women to identify prognostic determinant value collected at T0 and T24 by day 7.

**Men**							**Women**					
**Day-7 mortality**												
	**Univariate analysis**			**Multivariate analysis**			**Univariate analysis**			**Multivariate analysis**		
	OR	95% CI	*p*	OR	95% CI	*p*	OR	95% CI	*p*	OR	95% CI	*p*
**Lac T0**	1.11	1.02–1.18	<0.001	1.08	1.01–1.17	0.018	1.10	1.03–1.16	0.002	1.03	0.98–1.09	0.170
**SOFA Score T0**	1.31	1.19–1.44	<0.001	1.30	1.17–1.45	<0.001	1.39	1.24–1.56	<0.001	1.26	1.14–1.39	<0.001
**Age**	1.04	1.02–1.06	<0.001	1.03	1.01–1.07	0.001	1.04	1.02–1.06	<0.001	1.06	1.03–1.09	<0.001
**Day-7 mortality**												
	**Univariate analysis**			**Multivariate analysis**			**Univariate analysis**			**Multivariate analysis**		
	OR	95% CI	*p*	OR	95% CI	*p*	OR	95% CI	*p*	OR	95% CI	*p*
**Lac T24**	1.26	1.15–1.39	<0.001	1.25	1.07–1.61	0.002	1.25	1.14–1.37	<0.001	1.29	1.11–1.58	<0.001
**SOFA Score T24**	1.52	1.32–1.75	<0.001	1.49	1.31–1.87	<0.001	1.53	1.30–1.80	<0.001	1.35	1.17–1.69	0.001
**Age**				1.06	1.02–1.13	0.004				1.06	1.02–1.12	0.010

## Data Availability

The data presented in this study are available on request from the corresponding author. The data are not publicly available due to ethical and privacy restrictions regarding participant data.

## Supplementary Materials

The following supporting information can be downloaded at: https://www.mdpi.com/article/10.3390/ijms27093753/s1.
