# Supplementary material for: Sepsis in the Emergency Department: Different Faces, Same Fate? A Sex-Based Prognostic Analysis
Source: Int J Mol Sci. 2026 Apr 23;27(9):3753. doi: 10.3390/ijms27093753 (PMC13163694; doi:10.3390/ijms27093753)
Supplement: Supplementary file 1 [file ijms-27-03753-s001.zip › ijms-4219932-supplementary.pdf]

	T0				T24			
	All (n=1527)	Male (n=858)	Female (n=669)	p	All (n=1460)	Male (n=822)	Female (n=638)	p
Heart rate (b/min)	98±23	97±23	98±23	0.317	90±20	90±20	91±20	0.340
Systolic BP (mmHg)	105±29	104±28	105±30	0.661	107±27	107±25	107±28	0.814
Respiratory rate (a/min)	24±8	23±7	24±8	0.078	22±8	22±9	22±8	0.240
Body temperature (°C)	37±1.4	37.1±1.4	37±1.5	0.452	36.4±2.3	36.4±2.5	36.5±2	0.510
Glasgow Coma Scale	15[14-15]	15[14-15]	15[14-15]	0.230	15[15-15]	15[15-15]	15[15-15]	0.520
pH	7.40±0.1	7.41±0.1	7.39±0.1	0.016	7.39±0.1	7.40±0.07	7.38±0.08	<0.001
P/F ratio	278±125	271±126	286±123	0.023	266±117	259±113	275±123	0.028
HCO_3^- (meq/L)	22±8	22±10	22±6	0.769	21.±6	22±5	21±6	0.253
CO_2	35±14	34±14	36±14	0.045	37±13	36±15	38±10	0.045
Lactate (meq/L)	3.2±3	3.3±3	3.2±3	0.234	1.9±1.6	1.9±1.6	2.0±1.8	0.550
Systolic shock index s	1±0.5	1±0.5	1±0.4	0.769	0.9±0.4	0.9±0.4	0.9±0.4	0.612
Diastolic shock index	1.86±0.9	1.8±0.8	1.9±0.9	0.253	1.7±0.6	1.7±0.6	1.7±0.6	0.253
C reactive protein	137±125	132±125	145±125	0.263	186±115	180±113	193±118	0.264
Procalcitonin	35±75	38±75	33±75	0.440	40±113	40±74	41±150	0.791
Neutrophile/lymphocyte ratio	20.1±22	19.7±24	20.7±18	0.613	25±48	26±59	23±24	0.675
Platelet/ lymphocyte ratio	380±761	385±932	373±411	0.849	318±547	326±670	306±283	0.725
Platelets (/mm^3)	190±112	183±111	199±112	0.004	266±117	259±113	275±123	0.028

Table S1 Vital signs and lab parameters in the whole study population and based on gender, at the first evaluation and after 24 hours.

	T0						T24					
	**Day-7 mortality**											
	**Univariate analysis**			**Multivariate analysis**			**Univariate analysis**			**Multivariate analysis**		
	OR	95% CI	p	OR	95% CI	p	OR	95% CI	p	OR	95% CI	p
**Lac**	1.10	1.06-1.15	<0.001	1.06	1.01-1.11	0.008	1.26	1.18-1.34	<0.001	1.27	1.14-1.40	<0.001
**SOFA Score**	1.33	1.24-1.43	<0.001	1.32	1.21-1.42	<0.001	1.50	1.35-1.66	<0.001	1.43	1.26-1.62	<0.001
**Age**	1.04	1.02-1.05	<0.001	1.04	1.02-1.06	<0.001				1.06	1.03-1.06	<0.001
**Sex**	1.06	0.76-1.47	0.706	1.08	0.74-1.55	0.689				1.26	0.71-2.23	0.432
	**Day-28 mortality**											
	**Univariate analysis**			**Multivariate analysis**			**Univariate analysis**			**Multivariate analysis**		
	OR	95% CI	p	OR	95% CI	p	OR	95% CI	p	OR	95% CI	p
**Lac**	1.09	1.05-1.13	<0.001	1.05	1.01-1.10	0.007	1.22	1.14-1.30	<0.001	1.17	1.06-1.27	<0.001
**SOFA Score**	1.25	1.18-1.33	<0.001	1.25	1.18-1.34	<0.001	1.41	1.30-1.53	<0.001	1.39	1.26-1.53	<0.001
**Age**	1.03	1.02-1.04	<0.001	1.03	1.02-1.04	<0.001				1.05	1.02-1.07	<0.001
**Sex**	0.96	0.75-1.23	0.771	0.99	0.74-1.32	0.978				1.08	0.70-1.66	0.719

Table S2 Univariate and multivariate analysis in whole population

**T0**						
	FEMALE			MALE		
	Survivor (n=595)	Not survivor (n=74)	p	Survivor (n=762)	Not survivor (n=96)	p
P/F ratio	289±125	275±118	0.129	275±117	257±150	0.005
Lactate (meq/L)	2.9±1.5	3.9±2.1	0.001	3±2.8	4±3.7	<0.001
Systolic shock index s	1.01±0.4	1.07±0.4	0.177	1±0.5	1±0.4	0.084
Diastolic shock index	1.7±0.5	1.8±0.8	0.466	1.7±0.6	1.8±0.7	0.271
Neutrophile/lymphocyte ratio	21±19	18±16	0.544	21±19	15±14	0.328
Platelet/ lymphocyte ratio	361±350	408±490	0.495	409±356	322±309	0.808
SOFA	4[3-6]	5[4-7]	<0.001	5[3-6]	6[5-7]	<0.001
**T24**						
	FEMALE			MALE		
	Survivor (n=498)	Not survivor (n=140)	p	Survivor (n=642)	Not survivor (n=180)	p
P/F ratio	282±121	253±125	0.018	274±110	212±108	<0.001
Lactate (meq/L)	1.7±1,4	3.2±2.1	<0.001	1.7±1.7	2.4±2.3	<0.001
Systolic shock index	0.87±0.33	1.14±0.48	<0.001	0.9±0.3	1±0.4	<0.001
Diastolic shock index	1.8±0.8	2.3±1.7	<0.001	1.8±0.8	2.1±0.9	<0.001
Neutrophile/lymphocyte ratio	21±20	31±30	0.006	26±22	24±20	0.269
Platelet/lymphocyte ratio	289±261	389±342	0.100	317±322	351±335	0.140
SOFA	4[2-5]	6[4-7]	<0.001	4[3-6]	7[5-8]	<0.001

Table S3 Prognostic determinants among women and men

	Men						Women					
	**Day-28 mortality**											
	**Univariate analysis**			**Multivariate analysis**			**Univariate analysis**			**Multivariate analysis**		
	OR	95% CI	p	OR	95% CI	p	OR	95% CI	p	OR	95% CI	p
**Lac T0**	1.09	1.04-1.015	<0.001	1.07	1.01-1.14	0.014	1.08	1.02-1.14	0.004	1.04	0.98-1.12	0.128
**SOFA Score T0**	1.25	1.16-1.35	<0.001	1.27	1.17-1.38	<0.001	1.26	1.16-1.38	<0.001	1.24	0.98-1.12	<0.001
**Age**	1.03	1.01-1.04	<0.001	1.02	1.0-1.04	0.003	1.03	1.02-1.05	<0.001	1.04	0.02-1.07	<0.001
	**Day-28 mortality**											
	**Univariate analysis**			**Multivariate analysis**			**Univariate analysis**			**Multivariate analysis**		
	OR	95% CI	p	OR	95% CI	p	OR	95% CI	p	OR	95% CI	p
**Lac T24**	1.17	1.08-1.28	<0.001	1.09	0.95-1.31	0.160	1.26	1.15-1.38	<0.001	1.26	1.08-1.56	<0.001
**SOFA Score T24**	1.44	1.30-1.60	<0.001	1.42	1.30-1.64	<0.001	1.40	1.23-1.59	<0.001	1.35	1.16-1.59	<0.001
**Age**				1.04	1.02-1.08	0.002				1.06	1.03-1.10	<0.001

Table S4 Univariate and multivariate analysis in men and women to identify prognostic determinant value at T0 and T24 by day-28 mortality

Men							Women					
**Day-7 mortality**												
	**Univariate analysis**			**Multivariate analysis**			**Univariate analysis**			**Multivariate analysis**		
	OR	95% CI	p	OR	95% CI	p	OR	95% CI	p	OR	95% CI	p
**Lac T0**	1.14	1.05-1.21	<0.001	1.10	1.03-1.22	0.014	1.11	1.02-1.21	0.001	1.02	0.96-1.11	0.185
**SOFA Score T0**	1.41	1.27-1.52	<0.001	1.30	1.17-1.45	<0.001	1.46	1.21-1.53	<0.001	1.30	1.16-1.42	<0.001
**Age**	1.05	1.03-1.07	<0.001	1.03	1.01-1.07	0.001	1.06	1.02-1.10	<0.001	1.06	1.03-1.10	<0.001
**Day-7 mortality**												
	**Univariate analysis**			**Multivariate analysis**			**Univariate analysis**			**Multivariate analysis**		
	OR	95% CI	p	OR	95% CI	p	OR	95% CI	p	OR	95% CI	p
**Lac T24**	1.24	1.13-1.42	<0.001	1.25	1.10-1.63	0.003	1.26	1.15-1.38	<0.001	1.25	1.07-1.51	<0.001
**SOFA Score T24**	1.46	1.30-1.76	<0.001	1.49	1.31-1.87	<0.001	1.54	1.31-1.81	<0.001	1.42	1.21-1.75	<0.001
**Age**				1.06	1.03-1.14	0.006				1.05	1.01-1.11	0.012

**Day-28 mortality**												
	**Univariate analysis**			**Multivariate analysis**			**Univariate analysis**			**Multivariate analysis**		
	OR	95% CI	p	OR	95% CI	p	OR	95% CI	p	OR	95% CI	p
**Lac T0**	1.10	1.02-1.21	<0.001	1.10	1.02-1.19	0.028	1.11	1.02-1.17	0.004	1.01	0.96-1.06	0.370
**SOFA Score T0**	1.31	1.15-1.40	<0.001	1.33	1.18-1.46	<0.001	1.40	1.22-1.58	<0.001	1.26	1.14-1.39	<0.001
**Age**	1.05	1.03-1.07	<0.001	1.04	1.02-1.08	0.001	1.05	1.02-1.06	<0.001	1.06	1.03-1.09	<0.001
**Day-28 mortality**												
	**Univariate analysis**			**Multivariate analysis**			**Univariate analysis**			**Multivariate analysis**		
	OR	95% CI	p	OR	95% CI	p	OR	95% CI	p	OR	95% CI	p
**Lac T24**	1.25	1.14-1.38	<0.001	1.24	1.06-1.61	0.002	1.35	1.24-1.41	<0.001	1.29	1.11-1.58	<0.001
**SOFA Score T24**	1.45	1.31-1.65	<0.001	1.49	1.31-1.87	<0.001	1.67	1.40-1.91	<0.001	1.39	1.17-1.75	<0.001
**Age**				1.08	1.04-1.17	0.002				1.11	1.07-1.115	0.007

Table S5 Sensitive analysis on patients admitted with Sepsis 3 criteria (832 patients)
